# Supplementary material for: Effects of sodium nitrite reduction, removal or replacement on cured and cooked meat for microbiological growth, food safety, colon ecosystem, and colorectal carcinogenesis in Fischer 344 rats
Source: NPJ Sci Food. 2023 Oct 7;7:53. doi: 10.1038/s41538-023-00228-9 (PMC10560221; doi:10.1038/s41538-023-00228-9)
Supplement: Supplementary file 1 — Supplementary information [file 41538_2023_228_MOESM1_ESM.docx]

**Supplementary information**

-------

***Effects of sodium nitrite reduction, removal or replacement on cured and cooked meat for microbiological growth, food safety and colon ecosystem, and colorectal carcinogenesis in Fischer 344 rats***

-------

**1. Supplementary Results: impact of sodium nitrite concentrations and vegetable stock in a cooked ham model on fecal and urinary biomarkers of lipid peroxidation and on NOC formation**

The consumption of processed meats did not induce a significant difference between the 4 experimental groups (see Supplementary Fig. 1). The presence of sodium nitrite (40 or 80 mg/kg of meat) or vegetable stock (mimicking the intake of 40 or 80 mg of nitrites/kg of meat) in processed meats did not modify fecal (Supplementary Fig. 1A/TBARS) and global (Supplementary Fig. 1A/DHN-MA) lipid peroxidation biomarkers, the formation of total nitroso compounds (Supplementary Fig. 1B/ATNC) and nitrosylated iron (Supplementary Fig. 1B/FeNO) as well as fecal heme iron bioavailability (Supplementary Fig. 1C) in rats.

**Supplementary Fig. 1** **Impact of sodium nitrites levels and vegetable stocks in cooked ham models on fecal and urinary biomarkers of lipid peroxidation and NOCs formation**

**A** Lipid peroxidation measured as TBARS (MDA equivalents, mM) in fecal water and DHN-MA in urine of 24h (ng/vol of 24h). **B** Nitroso-compounds in fecal water measured as total NOCs (ATNC), as nitrosyl iron (FeNO), (nmol/gd of feces) **C**-Heme in fecal water (µM) Data were represented using scatter plots with bar (mean ± sem, n = 5, except when outliers are removed), * p ≤ 0.05; **p ≤ 0.01.

**2. Supplementary Results: biochemical characteristics of cooked ham models used for the CRC animal study**

|  | **Iron** | **Zinc** |
| --- | --- | --- |
| **PRE** | 1.15 ± 0.02 | 2.92 ± 0.20 |
| **Ni-120** | 1.25± 0.14 | 2.93 ± 0.19 |

**Supplementary Table 1 Quantification of total iron and zinc by ICP AES in PRE and Ni-120 cooked ham models, expressed in mg/kg**

Data were mean ± SD

The results presented in Supplementary Table 1 show an equivalent zinc concentration between the two cooked ham models.

|  | n | NDMA | | NMEA | | NDEA | | NDiBA | | NDBA | | NDMA + NDEA |  |
| --- | --- | --- | --- | --- | --- | --- | --- | --- | --- | --- | --- | --- | --- |
| Ni-120 | 3 | <1.0 | | <1.0 | | <1.0 | | <1.0 | | <1.0 | | <1 |  |
| VS | 3 | <1.0 | | <1.0 | | <1.0 | | <1.0 | | <1.0 | | <1 |  |
| PRE | 3 | <1.0 | | <1.0 | | <1.0 | | <1.0 | | <1.0 | | <1 |  |
| YE | 3 | <1.0 | | <1.0 | | <1.0 | | <1.0 | | <1.0 | | <1 |  |
|  |  | |  | |  | |  | |  | |  |  |  |

**Supplementary Table 2 Quantification of five volatile *N*-nitrosamines (µg/kg) in Ni-120 and the three alternatives (VS, PRE and YE).** *N*-Nitrosodimethylamine (NDMA), *N*-Nitrosomethylethylamine (NMEA), *N*-Nitrosodiethylamine (NDEA), *N*-nitrosodiisobutylamine (NDiBA), *N*-Nitrosodibutylamine (NDBA)

The results presented in Supplementary Table 2 show results below the quantification limit of the method.

**3. Supplementary Results: *Listeria monocytogenes* growth assay in sliced cooked ham model products**


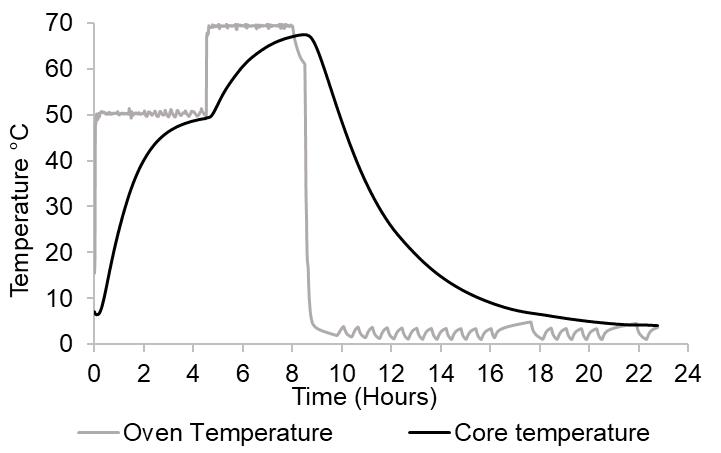


**Supplementary Fig. 2 Heat treatment kinetics** obtained in ambience of the oven chamber (grey) and into the cooked ham model product (black) for the first experimental trial as an example

| **Recipe** | **pH** | |  | **a_w_** | |  | **NaCl (%)** | |  | **NaNO_2_ (mg/kg)** | |  | **NaNO_3_ (mg/kg)** | |  | **TBARS (mg MDA eq /kg)** | |  | **Red tint angle °** | |
| --- | --- | --- | --- | --- | --- | --- | --- | --- | --- | --- | --- | --- | --- | --- | --- | --- | --- | --- | --- | --- |
|  | **D0** | **D49** |  | **D0** | **D49** |  | **D0** | **D49** |  | **D0** | **D49** |  | **D0** | **D49** |  | **D0** | **D49** |  | **D0** | **D49** |
| **Ni-120** | 6.12  ± 0.047 | 5.96  ± 0.230 |  | 0.979  ± 0.004 | 0.980  ± 0.008 |  | 1,78  ± 0,05 | *ND* |  | 34.8  ± 7.1 | 8.4  ± 0.15 |  | 18.5  ± 2.44 | 33.3  ± 2.45 |  | *ND* | 0.312  ± 0.40 |  | 48.4  ± 3.03 | 48.4  ± 3.94 |
| **Ni-90** | 6.12  ± 0.043 | 5.88  ± 0.227 |  | 0.978  ± 0.003 | 0.979  ± 0.009 |  | 1,77  ± 0,06 | *ND* |  | 19.2  ± 6.11 | 3.2  ± 1.85 |  | 12.8  ± 1.60 | 23.1  ± 5.28 |  | *ND* | 0.321  ±0.035 |  | 48.4  ± 3.52 | 50.5  ± 2.86 |
| **Ni-0** | 6.11  ± 0.069 | 5.92  ± 0.231 |  | 0.98  ± 0.003 | 0.978  ± 0.006 |  | 1,8  ± 0,05 | *ND* |  | <1.5 | <1.5 |  | <6.9 | <6.9 |  | *ND* | 1.613  ±0.160 |  | 19.0  ± 2.36 | 12.3  ± 3.46 |
| **VS** | 6.11  ± 0.073 | 5.86  ± 0.219 |  | 0.975  ± 0.003 | 0.982  ± 0.005 |  | 1,91  ± 0,06 | *ND* |  | 15.4  ± 5.88 | <1.5 |  | 10.6  ± 5.58 | 17.7  ± 0.74 |  | *ND* | 0.300  ± 0.035 |  | 45.6  ± 3.93 | 49.1  ± 2.92 |
| **PRE** | 6.07  ± 0.090 | 5.84  ± 0.087 |  | 0.975  ± 0.005 | 0.977  ± 0.006 |  | 2,04  ± 0,07 | *ND* |  | <1.5 | <1.5 |  | 7.4  ± 0.38 | <6.9 |  | *ND* | 0.316  ±0.045 |  | 48.6  ± 3.43 | 48.0  ± 4.6 |
| **YE** | 6.10  ± 0.046 | 5.99  ± 0.300 |  | 0.976  ± 0.002 | 0.978  ± 0.004 |  | 2,07  ± 0,08 | *ND* |  | <1.5 | <1.5 |  | <6.9 | <6.9 |  | *ND* | 2.977  ±1.320 |  | 27.5  ± 2.54 | 16.0  ± 4.26 |

**Supplementary Table 3** **Physicochemical properties of the cooked ham model products** from the different recipes (mean values ± SD obtained from all 3 independent experiments).

| **Recipe** | **Lactic acid bacteria (Log_10_ CFU/g)** | |
| --- | --- | --- |
|  | **D0** | **D49** |
| **Ni-120** | 0.0 ± 0.00 | 7.8 ± 0.67 |
| **VS** | 0.4 ± 0.65 | 7.8 ± 0.87 |
| **PRE** | 0.6 ± 1.00 | 8.2 ± 0.38 |
| **YE** | 0.0 ± 0.00 | 7.9 ± 0.98 |
| **Ni-90** | 0.3 ± 0.50 | 8.2 ± 1.16 |
| **Ni-0** | 0.4 ± 0.61 | 7.1 ± 1.14 |

**Supplementary Table 4** **Lactic acid bacteria populations** (Log_10_ CFU/g) enumerated in the cooked ham model products from the different recipes (mean values ± SD obtained from all 3 independent experiments).

Cooked ham model samples used in the microbiological assays exhibited typical pH (mean values from 6.07 to 6.12 regardless of the recipes) and a_w_ (mean values from 0.975 to 0.980 regardless of the recipes) values at D0. The a_w_ of the products remained steady during the 49-day storage whereas the pH slightly decreased (minimal mean value of 5.84 units regardless of the recipe). The proliferation of lactic acid bacteria (Supplementary Table 4) can be associated to the decrease in the measured pH levels. All pH and a_w_ values measured during the present study were permissive to the growth of *L. monocytogenes* (ANSES, 2021). The nitrate salt, nitrite salt and NaCl levels measured were consistent with the additive doses in corresponding recipes (Table 3 and Supplementary Table 3).

**4*.* Supplementary Results: test of interference with polyphenols on N-nitroso compounds (NOC) assay in fecal water**


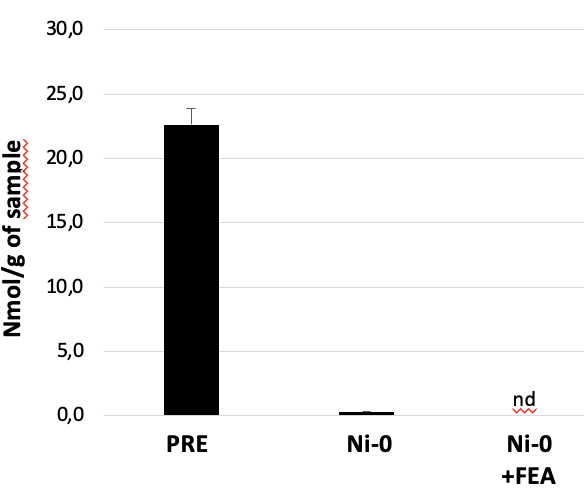


**Supplementary Fig. 3 ATNC concentration in fecal water** from PRE ham fed rats, from Ni-0 ham fed rats and fecal water of from Ni-0 ham fed rats supplemented in epigallocatechine gallate, ferulic acid and ascorbic acid (Ni-0 + FEA). Mean values ± SD

This assay, independent of the analyses presented in Fig. 6 of the main manuscript, confirmed a high presence of ATNC in the PRE fecal water and their absence in the Ni-0 fecal water. We also demonstrated that the overloading of Ni-0 fecal water with polyphenols and ascorbic acid present in the PRE formulation did not interfere with the assay and did not generate false positive results with our NOC analysis method.

**5*.* Supplementary Results: test of interference with polyphenols on cooked ham model *N*-nitroso compounds (NOC) assay**

|  | Total heme iron mM | Fe NO mM | % Fe-NO |
| --- | --- | --- | --- |
| Ni-0 | 0.14 ± 0.013 | 0.0012 ± 0.000 | 0.5 |
|  |  |  |  |
| ECGC 5 | 0.11 ± 0.01 | 0.0014 ± 0.000 | 1.3 |
|  |  |  |  |
| ECGC 10 | 0.11 ± 0.007 | 0.0023 ± 0.0006 | 2.2 |
|  |  |  |  |
| ECGC 20 | 0.12 ± 0.004 | 0.0049 ± 0.0002 | 4.1 |
| p < 0.05 | NS | * | * |

**Supplementary Table 5** **Total heme iron and nitrosyl heme iron (FeNO)** in Ni-0 ham (Ni-0) and the same ham loaded with different concentrations of epigallocatechine gallate (EGCG) (5 mg/mL, 10 mg/mL, 20 mg/mL).

This assay showed interference in the determination of nitrosyl heme by colorimetric method (Hornsey) when EGCG is added, while it was not the case for total heme iron (Supplementary Table 5).

|  | Total heme iron mM | FeNO mM | % Fe- NO |
| --- | --- | --- | --- |
| Ni-0 | 0.13 ± 0.02 | 0.0018 ± 0.0004 | 1.1 |
|  |  |  |  |
| Ni-0 + FEA | 0.21 ± 0.01 | 0.0217 ± 0.00005 | 10.6 |

**Supplementary Table 6** **Total heme iron and nitrosyl heme iron (FeNO)** in Ni-0 ham (Ni-0) and the same ham loaded with a mix of epigallocatechine gallate, ferulic acid and ascorbic acid (Ni-0 + FEA). (mean value ± SD)

This assay showed interference in the determination of total heme iron and nitrosyl heme by colorimetric method (Hornsey) when a mix of epigallocatechine gallate, ferulic acid and ascorbic acid was added. The total heme iron is over estimated by 60% while for nitrosyl iron the overestimation is multiply by 8. Lastly the % of nitrosyl heme is approximately overestimated by 10 (Supplementary Table 6).

|  | NO_2_ mg/kg | NO_3_ mg/kg | RSNO mg/kg | RNNO mg/kg |
| --- | --- | --- | --- | --- |
| Ni-0 | 0.05 ± 0.04 | 3.76 ± 0.09 | 0.23 ± 0.08 | 0.06 ± 0.04 |
|  |  |  |  |  |
| Ni-0 + FEA | 0 ± 0 | 1.97 ± 0.24 | 0.05 ± 0.04 | 0.33 ± 0.10 |

**Supplementary Table S** **Colorimetric determination of NO_2_, NO_3_, RSNO and RNNO** according to Bonifacie et al 2021^3^ using the Griess reagent, in Ni-0 ham (Ni-0) and the same ham loaded with a mix of epigallocatechine gallate (EGCG), ferulic acid and ascorbic acid (Ni-0 +FEA). 1g of Ni-0 ham was grounded with 1 mL of water (Ni-0) and 1g of Ni-0 ham was grounded in 1mL solution containing 100 µL d'EGCG 20 mg/mL in H_2_O; 150µL ferulic acid 53 mg/mL in ethanol; 100 µL ascorbic acid 50 mg/mL in water; 650 µL H_2_O (Ni-0 + FEA). Then nitroso-compounds were quantified as described previously^3^ in section 2.1.3.

This assay did not show any interference in the determination of NO_2_, NO, RSNO, RNNO by the colorimetric method of Griess when a mix of epigallocatechine gallate, ferulic acid and ascorbic acid was added. The level of RSNO and RNNO even significantly different were under the threshold sensitivity of 1 mg/kg (Supplementary Table 7).

**6*.* Supplementary Results: colon mucosa gene expression assays on samples of the 100days-study with different concentrations of sodium nitrite (0, 90 and 120 mg/kg) or sodium nitrite alternatives in cooked ham models.**

**Supplementary Table 8 Primer sequences for RT-qPCR analysis.**

Reduction or removal of sodium nitrite concentration had low or no impact on colon mucosal detoxification activities. The most striking effect, although not significant, is the dose-dependent increase in the expression of Nqo1, and trends to a decrease for Gsta4, Akr1b10, Cbr1, Sod1 in the case of sodium nitrite removal (Supplementary Fig. 4).

The effect of the alternatives appeared to be weaker with only one notable and significant effect of YE cooked ham model that induced a significant decrease in the expression of Hmox1 compared to cooked ham models VS and PRE and a significant increase in the expression of Cox2 compared to the reference cooked ham model Ni-120 and VS (Supplementary Fig. 5).

**Supplementary Fig. 4 Impact of sodium nitrite concentrations in cooked ham models (0 *vs* 90 *vs* 120 mg/kg) on gene** **expression in rat colon mucosa.** Data were represented using scatter plots with bar (mean ± sem, n = 12, except if outliers are removed), significance was determined by a Kruskal-Wallis followed by Dunn’s mean comparison test.* p ≤ 0.05. Genes full names are shown in Supplementary Table 8.

**Supplementary Fig. 5 sodium nitrite alternative effect (Vegetable Stock (VS), Polyphenol-rich Extract (PRE), Lallemand solution (YE) on gene** **expression in rat colon mucosa.** Data were represented using scatter plots with bar (mean ± sem, n = 12, except if outliers are removed), significance was determined by a Kruskal-Wallis followed by Dunn’s mean comparison test.* p ≤ 0.05. Genes full names are shown in Supplementary Table 8.

# 7. Supplementary Results: assessment of 8-isoprostane (8-isoPGF2α) in urinary samples of the 100 days-study with different level of sodium nitrite (0, 90 and 120 mg/kg) or alternatives to sodium nitrite in cooked ham models.

Modification of the sodium nitrite levels (Supplementary Fig. 6A) or sodium nitrite substitution by alternatives (Supplementary Fig. 6B) had no effect of urinary excretion of 8-isoPGF_2α_ per 24h.

******

**Supplementary Fig. 6** **Impact of sodium nitrite concentrations and alternatives on urinary 8-isoPGF_2α_ (pg/24h)**

**A-**Impact of sodium nitrite concentrations in processed meats (0 *vs* 90 *vs* 120 mg/kg). **B**- Impact of alternatives (Vegetable Stock (VS), Polyphenol-rich Extract (PRE), Lallemand solution (YE)). Data were represented using scatter plots with bar (mean ± sem, n = 12, except if outliers are removed). Data were analyzed with ANOVA followed by Tukey’s mean comparison.

# 8. Supplementary Results: abundance variation of fecal bacterial communities displaying dose effects and/or changes in response to alternatives to sodium nitrite in cooked ham model.

***7.1* Supplementary Table 9 Taxonomic affiliations of agglomerated at the genus rank**

**A** Taxonomic affiliation of clusters agglomerated at the genus rank significantly affected by the sodium nitrite content in ham-based diets (Deseq2, Padj≤0.05).

**B** Taxonomic affiliation of clusters agglomerated at the genus rank significantly affected by the alternative to nitrites in ham-based diets (Deseq2, Padj≤0.05).

******

***7.2 Supplementary Figures***

**
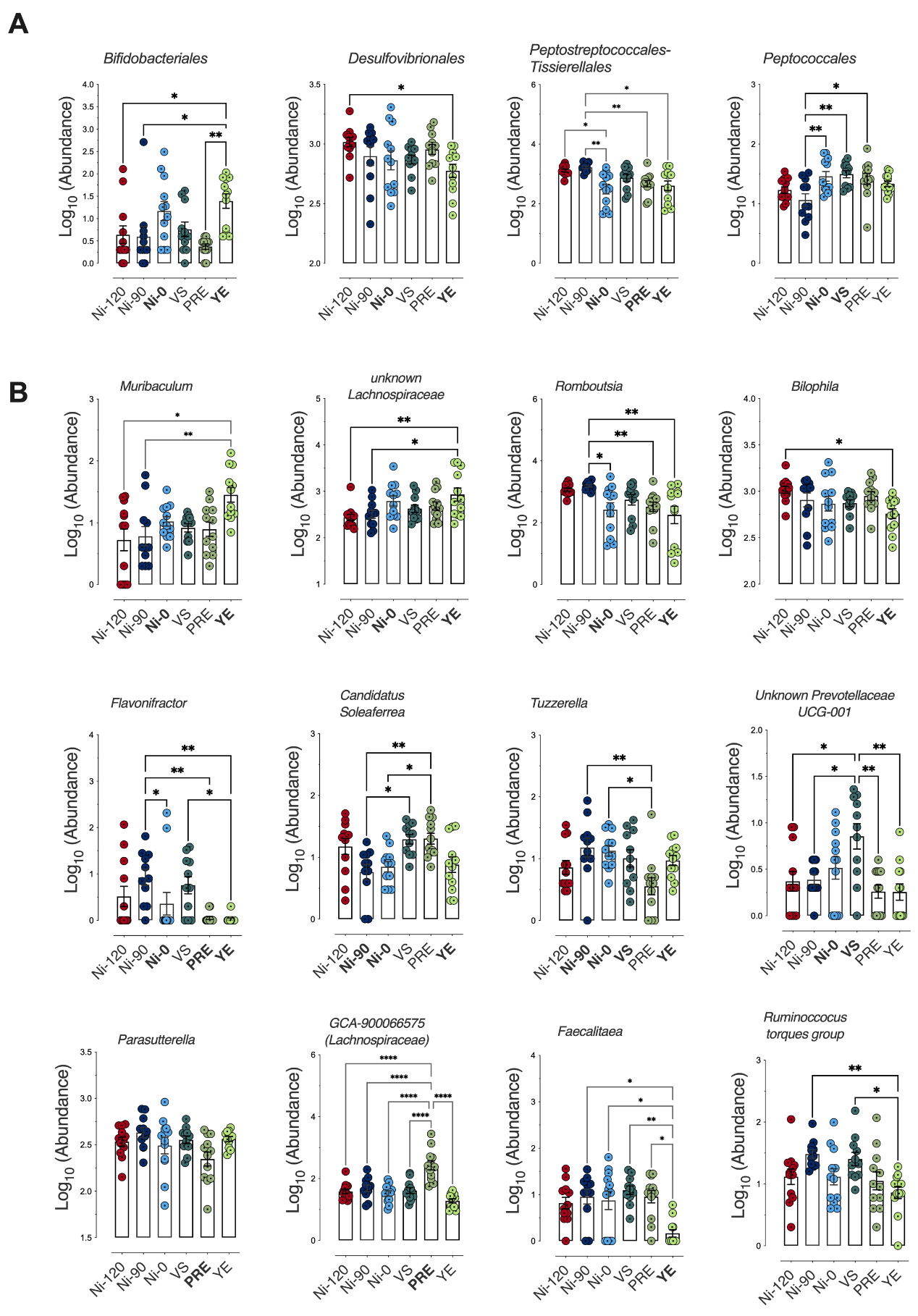
**

**Supplementary Fig. 7** **Impact of sodium nitrite concentrations and sodium nitrite alternatives on fecal microbiota of rats.** Normalized Log_10_ abundances at the order level (**A**) and genus level (**B**) resulted from agglomeration of OTUs. Data were represented using scatter plots with bar (mean ± sem), significance was determined by a Kruskal-Wallis followed by Dunn’s mean comparison test. * p ≤ 0.05; **p ≤ 0.01, ** p ≤ 0.01, ***p ≤ 0.001, ****p ≤ 0.0001.
